# Ovarian inguinal hernia – a possibility in MURCS syndrome

**DOI:** 10.1186/s13048-021-00869-y

**Published:** 2021-09-03

**Authors:** Rahul Saini, Lovenish Bains, Tarangpreet Kaur, Pawan Lal, Veer Pal, Mohd Yasir Beg, Daljit Kaur

**Affiliations:** 1 Department of Surgery, Maulana Azad Medical College, New Delhi, India; 2 Department of Obstetrics and Gynecology, All India Institute of Medical Sciences, New Delhi, India; 3 Department of Transfusion Medicine, All India Institute of Medical Sciences, Rishikesh, Uttarakhand India

**Keywords:** Ovary, Inguinal hernia, Mayer-Rokitansky-Kuster-Hauser (MRKH) syndrome, Mullerian agenesis, Renal agenesis, Cervico-thoracic Somite abnormalities (MURCS), Karyotype

## Abstract

**Background:**

Inguinal hernia containing ovary and fallopian tube can be found in paediatric population and is a rare finding in women of reproductive age group. Most of the cases are associated with congenital abnormalities of the female genital tract.

**Case presentation:**

A 20 year old female presented with right reducible inguinal hernia, primary amenorrhea and normal secondary sexual characteristics. Clinical examination revealed scoliosis with convexity towards left side, prominence of left rib cage with Sprengel deformity and right sided heart sounds. Ultrasound of the inguinal swelling revealed right ovary within the hernial sac, Chest X-ray revealed right lung collapse and dextrocardia. Further Magnetic resonance imaging (MRI) of pelvis revealed inguinal hernia with right ovary as its content, normal left ovary and absent uterus. Computed tomography (CT) revealed complete collapse of right lung with compensatory left lung hyperinflation and absent right kidney. Karyotyping of the patient was normal, 46XX. A diagnosis of MURCS syndrome with right ovarian hernia was made. The hernia was surgically managed with repositioning of ovary and fallopian tube into the pelvis.

**Discussion:**

Ovary in inguinal hernia is rare in women of reproductive age group. MRKH syndrome, a mullerian duct anomaly, is the congenital aplasia of uterus and upper two-thirds of vagina in a female with normal ovaries, fallopian tube, secondary sexual characteristics and 46XX karyotype. MURCS is a subtype of MRKH type 2 having mullerian duct agenesis with renal, cardiac, muscular & vertebral defects. General physical examination and primary investigations if yields abnormal findings; the patient must undergo an array of investigations to rule out MRKH/MURCS, or other congenital abnormality. Early diagnosis is essential to prevent its incarceration or torsion. The primary treatment of ovary in inguinal hernia is repositioning the ovary and fallopian tube back to pelvis to preserve fertility and repair of inguinal hernia. A multidisciplinary team is required to deal with various abnormalities present in a patient with MURCS.

## Introduction

Inguinal hernia is one of the most common surgery done worldwide [1]. The usual contents of inguinal hernia are omentum and small intestines. But sometimes its contents surprise the operating surgeon like sigmoid colon, caecum, appendix, urinary bladder, uterus etc [2]. An incomplete closure of the canal of nuck causes the development of inguinal hernia in females usually containing ovaries, fallopian tubes and uterus as contents. Inguinal hernias containing ovary occurs rarely in adult females [3]. A recent systematic search of 17 cases of ovarian inguinal hernia in women of reproductive age group analysed their presentation and treatment [4]. We report an unusual case of MURCS syndrome associated with inguinal hernia with ovary and fallopian tube as its content.

## Case presentation

A 20-year-old unmarried female presented to the surgery outpatient department with complaint of a right inguinal swelling since 2 years of age, which was reducible. Parents took an opinion about the swelling in her childhood but never followed it. On examination, a firm swelling of size 3 × 4 cm was present on right side which was reducible and showed cough impulse consistent with right sided inguinal hernia.

On general examination of the patient, scoliosis was present clinically with convexity towards left side. The left side rib cage was more prominent than the right side. Left sided Sprengel deformity was present with webbed neck and increased carrying angle of right elbow. Chest examination revealed right sided heart sounds, but no murmurs were heard. There were diminished breath sounds on the right side of chest, but normal breath sounds on the left side.

**Fig. 1:**
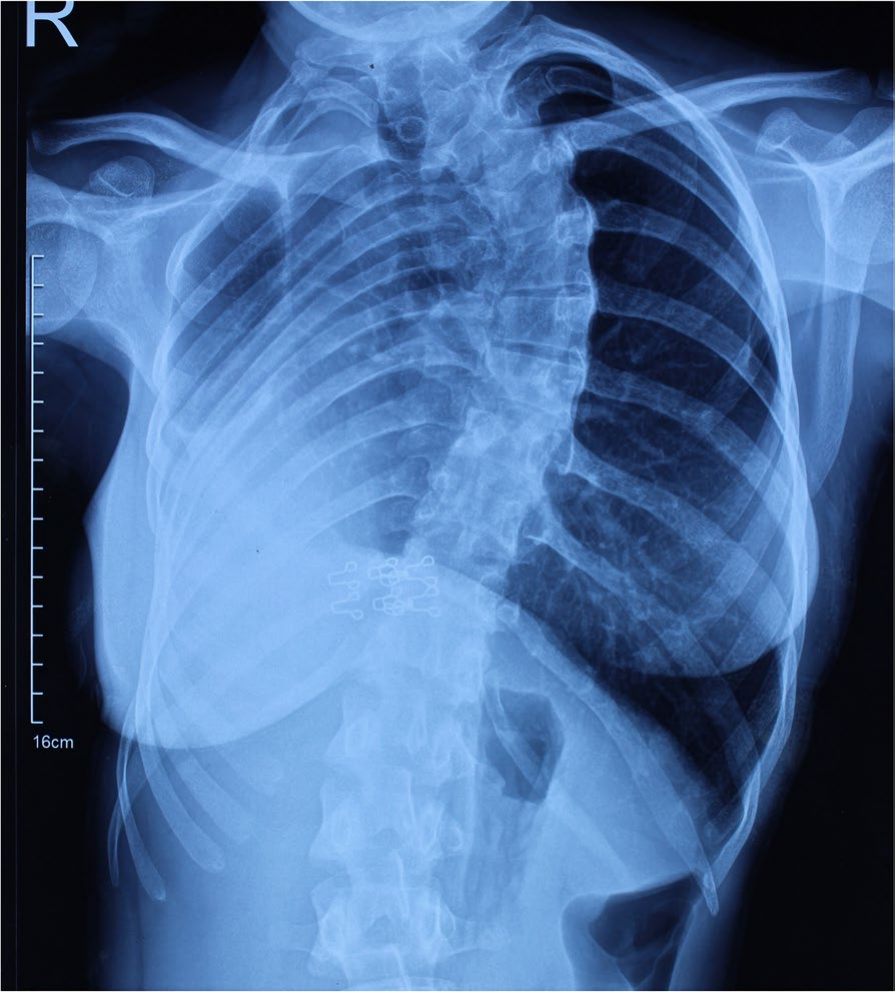
CXR showing right lung collapse and dextrocardia

Patient also complained of primary amenorrhea after attainment of puberty. On examination, vaginal length was approximately 2 – 2.5 cm. On per rectal examination, uterus was not felt anteriorly. Bilateral breast and pubic hair were in tanner stage 4 of sexual development. Ultrasound of the inguinal swelling revealed right ovary within the hernial sac of size 3.1 × 3.3 cm with normal left ovary in the pelvis. Chest X-ray revealed right lung collapse (Fig. 1). Echocardiography revealed dextrocardia with normal heart chambers and valves, and ejection fraction of 60%. The patient seemed to have some congenital syndrome by examination and above mentioned findings and hence was subjected to further investigations in consultation with other departments.

**Fig. 2:**
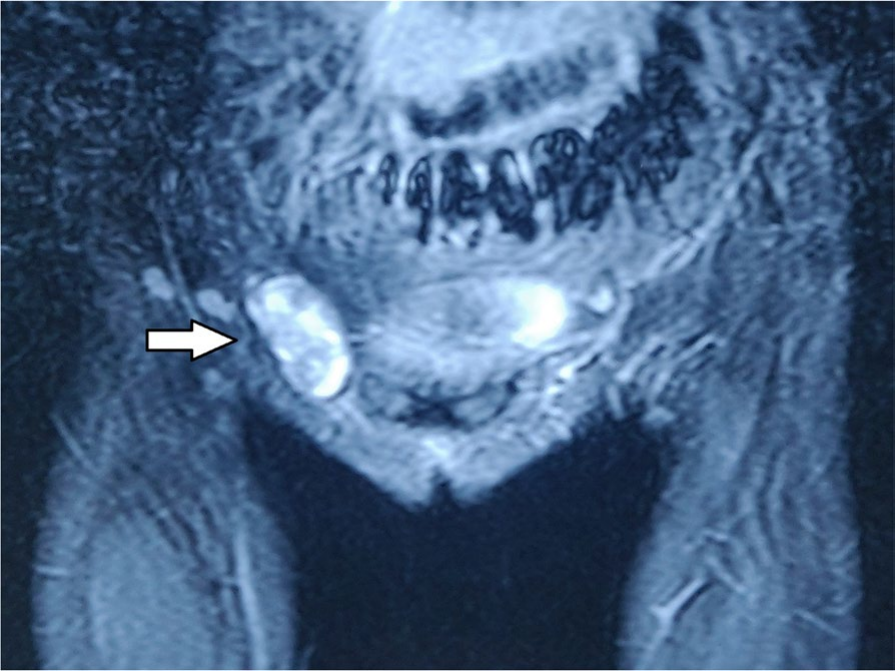
MRI (coronal plane) showing ovary in inguinal region

**Fig. 3:**
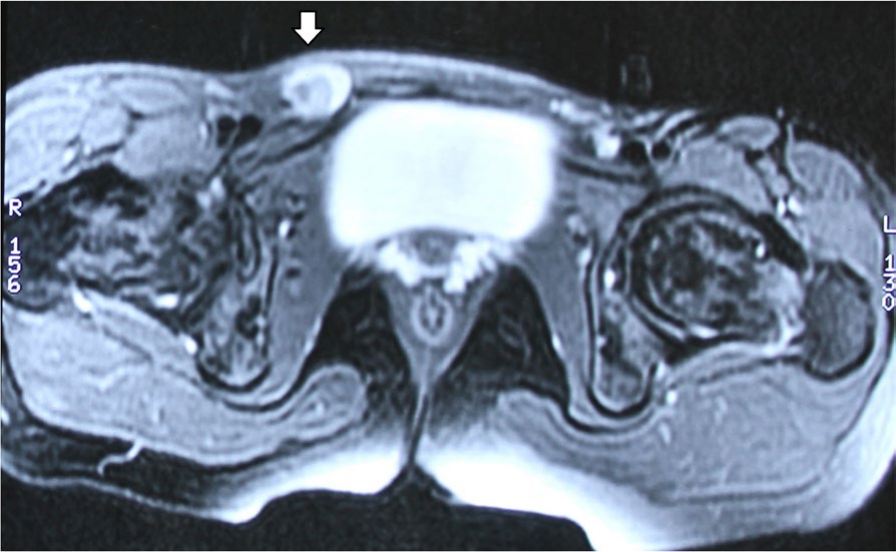
MRI- transverse plane

**Fig. 4:**
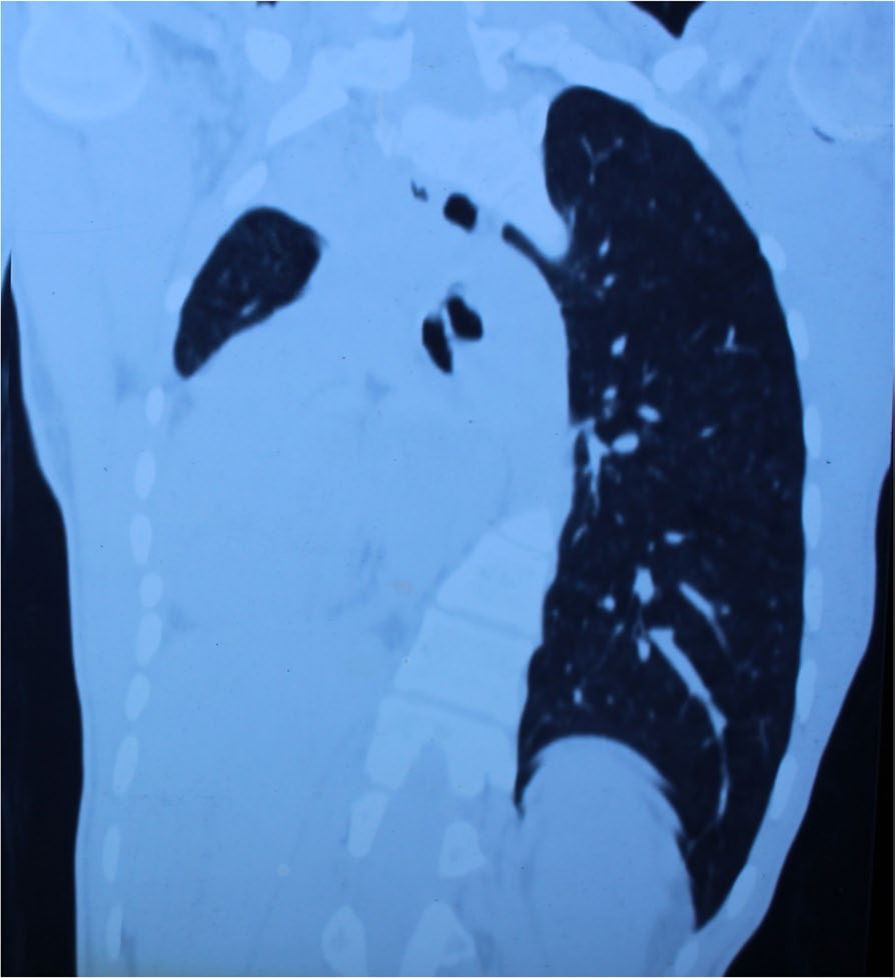
CT showing right lung collapse

**Fig. 5:**
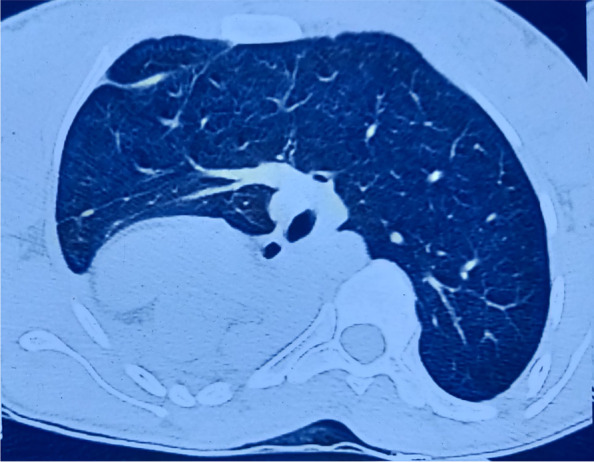
CT (transverse plane) showing compensated left lung and dextrocardia

**Fig. 6:**
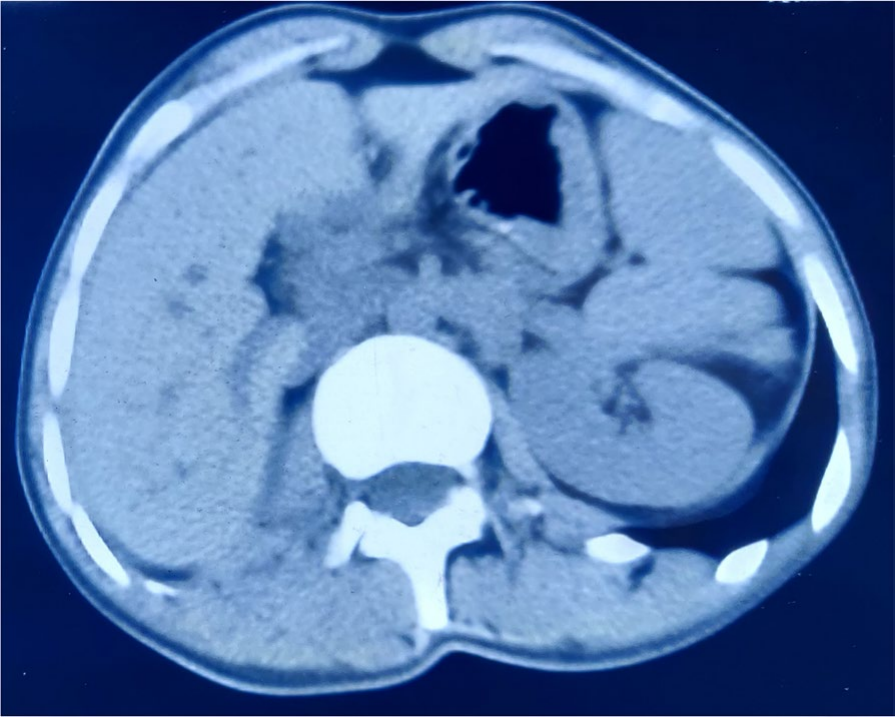
CT showing absent kidney

**Fig. 7:**
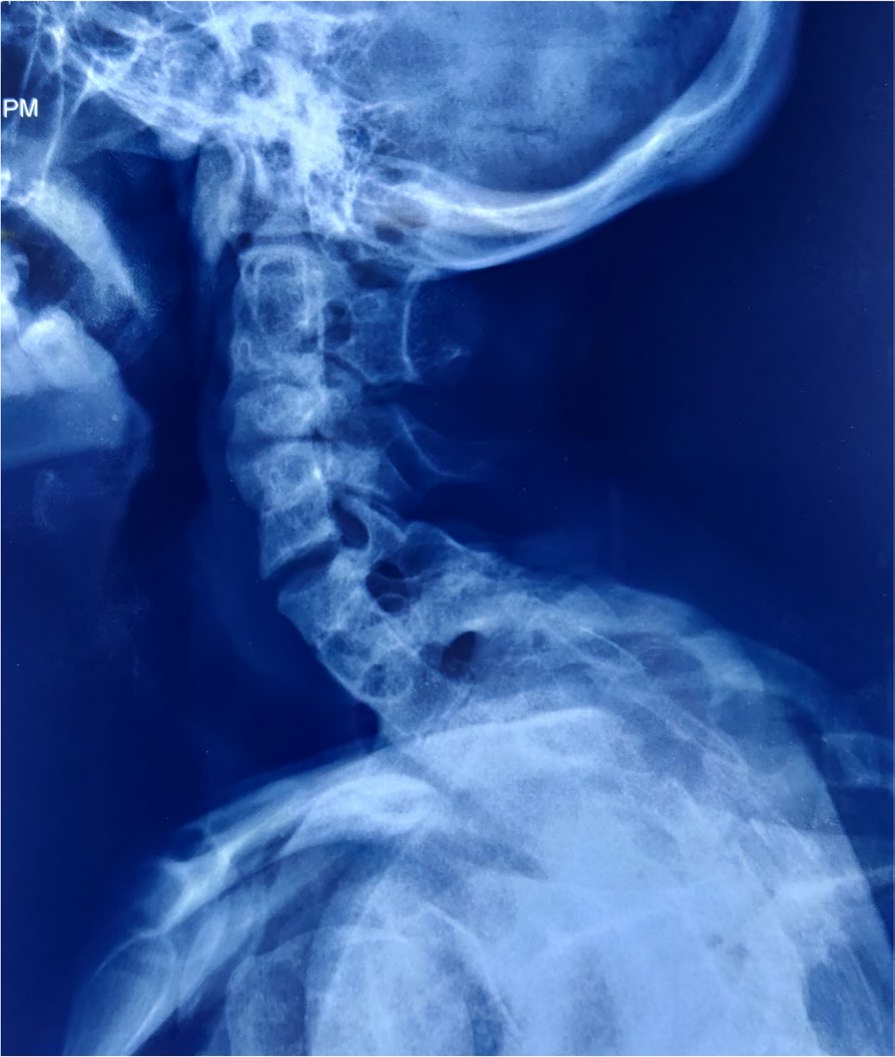
X ray showing fused cervical spine

Magnetic resonance imaging (MRI) of pelvis revealed indirect inguinal hernia with right ovary as its content, normal left ovary and absent uterus (Fig. 2, 3). A suspicion of MRKH syndrome was made based on it. Computed tomography (CT) revealed complete collapse of right lung with compensatory left lung hyperinflation and absent right kidney (Fig. 4, 5, 6). Pulmonary function test suggested severe obstruction with restriction. X-ray spine revealed fusion of the posterior elements of 2^nd^ to 7^th^ cervical vertebral bodies associated with scoliotic deformity, maximum at 5^th^ thoracic vertebra, with convexity towards left side (Fig. 7). Karyotyping of the patient was normal, 46XX.

**Fig. 8:**
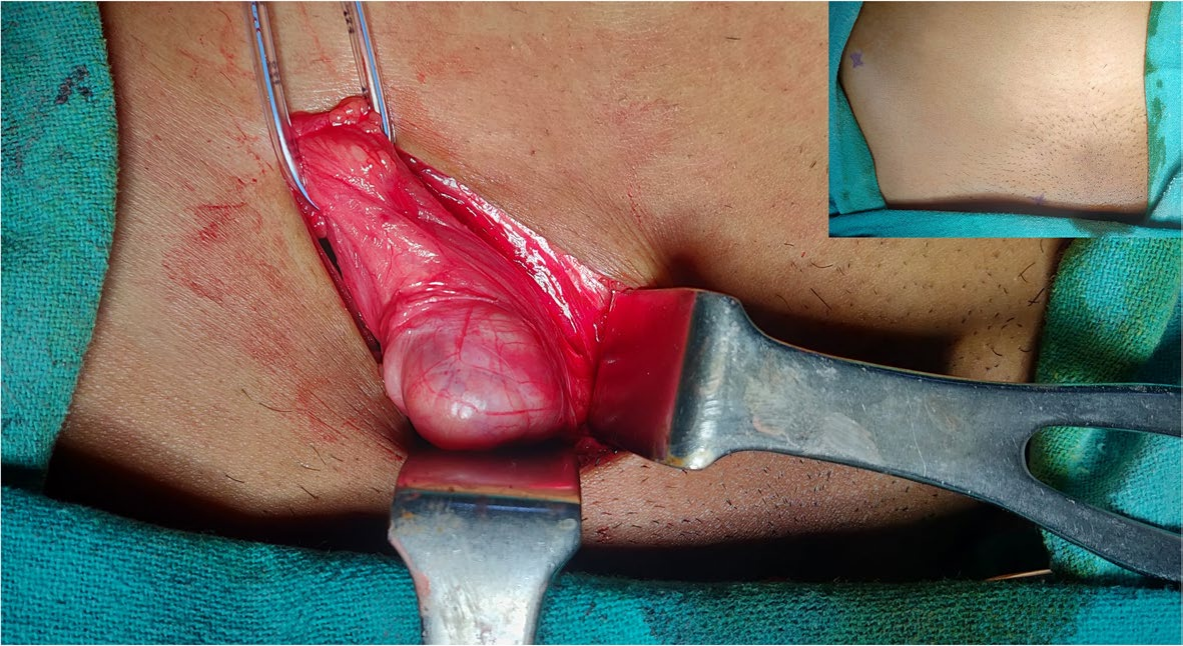
Intra operative picture showing ovary within sac and reduced inguinal swelling (inset)

**Fig. 9:**
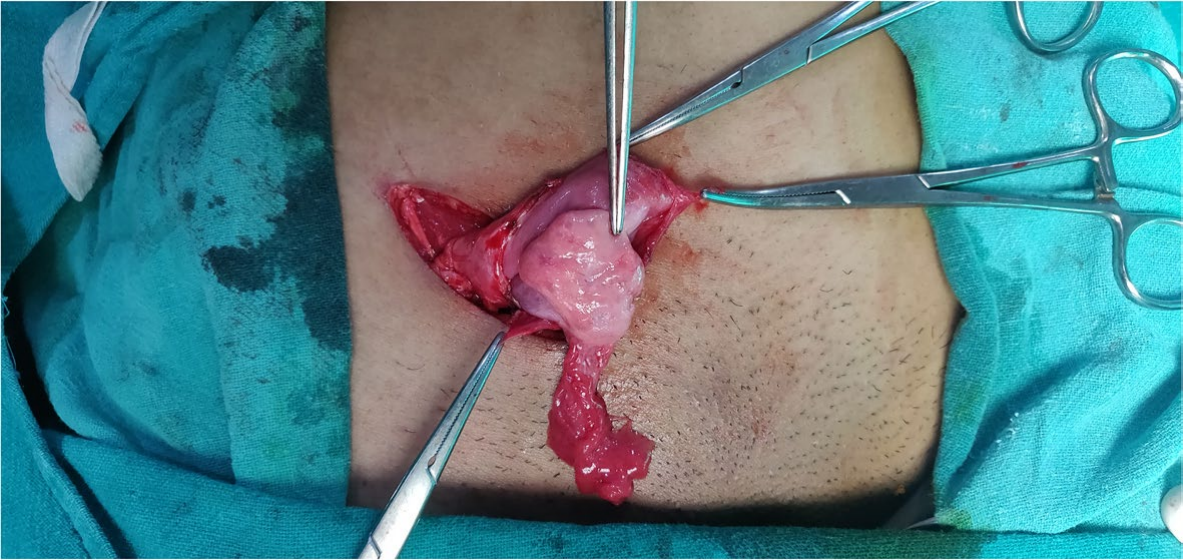
Picture showing right ovary and fallopian tube

A diagnosis of MURCS association with unilateral lung agenesis with right inguinal ovarian hernia was made. Blood investigations revealed haemoglobin of 12.8 gm/l, platelets 2.6 lacs/mm [3], blood urea 21 mg/dl and serum creatinine 0.8 mg/dl. The patient underwent elective open hernia repair wherein, the intraoperative findings revealed indirect hernial sac containing right ovary with fallopian tube. (Fig. 8, 9) The contents were repositioned back into the pelvic cavity and the hernia repaired by modified Bassini’s technique. Postoperative period was uneventful with the patient being discharged on day 3. The patient is healthy up to 1 year of follow up and is being treated by a multidisciplinary team.

## Discussion

Mayer-Rokitansky-Küster-Hauser (MRKH) syndrome has a prevalence of 1 in 4000–5000 female patients [5]. MRKH syndrome, a Mullerian duct anomaly, is the congenital aplasia of uterus and upper two-thirds of vagina in a female with normal ovaries, fallopian tube, secondary sexual characteristics and 46XX karyotype [6]. It is inherited as an autosomal dominant trait with an incidence of 1 in 4500 female births [5, 7]. There are two forms of MRKH syndrome. Type 1, accounting for 44% of MRKH syndromes, is the isolated Mullerian abnormality, i.e., congenital absence of uterus and vagina. Type 2, accounting for 56% of MRKHS, has Mullerian duct agenesis with renal, cardiac, muscular & vertebral defects. MURCS (Mullerian agenesis, Renal agenesis, Cervico-thoracic Somite abnormalities) is a subtype of MRKH type 2, with predefined multi-organ abnormalities. It accounts for 16% of MRKH type 2 cases [8]. Such individuals have normal female body pattern with normal thelarche and adrenarche.

Inguinal hernia containing ovary and fallopian tube is generally found in paediatric population as groin swellings, that accounts for 6–7% of inguinal hernias in this age group [9–11]. In one series by Ein et al., the incidence in more than 1000 females was 15% but 76% were younger than 2 years [12]. Almost 30% of all reported cases are related to adolescents or women of reproductive age [13]. In another series of 1,950 groin hernia patients, the overall incidence of inguinal hernia containing ovary as a content was seen in < 2.9% [3]. Association between MRKH type I syndrome and inguinal hernia with ovary as a content has been reported in few isolated case reports however delayed presentation in adult age with MURCS is very rare [4]. Such cases also have abnormal genital development. Such presentations are found uncommonly in adult females [10]. Ovarian torsion and infarction are seen in 2–33% of patients presenting as ovarian hernia [14]. The incidence of incarceration (acute or chronic) was 9% [12]. Early diagnosis and management are of prime importance to restore fertility in such patients as these ovaries are more prone to torsion and infarction.

Female adnexa might get entrapped in the inguinal canal and present as inguinal hernia. Various anatomical reasons exist for its occurrence, like, very short & less oblique inguinal canal or presence of a peritoneal pouch, similar to processus vaginalis, called as canal of nuck [14].

Various theories were given to explain the development of ovarian hernia in females. According to a hypothesis given by Thomson, non-fusion of mullerian ducts lead to hypermobility of ovaries, thereby increasing the chances of entrapment of adnexa in the inguinal canal [15]. Fowler, on the other hand, hypothesised that the congenitally elongated ovarian ligaments were the main cause of inguinal hernia with ovary in the canal [16]. Another theory suggested that the weakness of broad ligament or ovarian ligament along with increase in the intra-abdominal pressure, contributed to herniation of ovary in inguinal canal [17]. Multiparity can cause lengthening of the broad ligament, ovarian or suspensory ligaments, increasing the chance of entrapment of adnexa in inguinal canal [17].

Normal ovary in a hernial sac is seen as a hypoechoic structure with variable sized sonolucent cysts on ultrasonography. Transabdominal ultrasound reveals the absence of ovary in the pelvic cavity. MRI pelvis on T2 weighted sequences demonstrates anatomy of uterus. Uterine aplasia is best diagnosed on T2 sagittal image. Normal vagina is seen as a structure of intermediate signal intensity between the base of bladder and urethra anteriorly and anal canal posteriorly. Vaginal agenesis is best demonstrated on axial images of MRI [18].

In our patient, MRI revealed right side ovary as a content of right inguinal hernia sac with absence of uterus and normal left ovary within the pelvis. A battery of investigations was done to look for other congenital malformations, in order to formulate the diagnosis of MURCS syndrome. According to a recent systematic search of literature of women aged > 18 years for ovarian hernia found a total 17 cases, 15 of which presented as groin swelling as tender irreducible lump requiring urgent intervention. 14 cases underwent open surgical repair, 2 had laparoscopic repair and 1 didn’t undergo surgery [4] The surgery was repositioning of structures (in 11), oophorectomy (in 2), and salpingo-oophorectomy (in 2) with repair of the hernia [6].

In our case, we performed modified open Bassini’s technique without mesh placement. Intraoperative findings included right ovary within the hernial sac along with right fallopian tube. The right ovary with fallopian tube was repositioned back into the pelvic cavity. Treatment of MRKH/MURCS syndrome requires a multipronged approach. Psychological counselling is required to counter the emotional aspects of patients. Healthy sexual relationship with in-vitro fertilization and surrogacy is possible. Manual dilatation of vagina using dilators and surgical creation of neo-vagina are options given to patients for performance of sexual intercourse.

## Conclusion

Ovary and fallopian tube in inguinal hernia is rare in women of reproductive age group. General physical examination and primary investigations if yields abnormal findings; the patient must undergo an array of investigations to rule out MRKH/MURCS syndrome, or other congenital abnormality. Early diagnosis is essential to prevent its incarceration or torsion. The primary treatment of ovary in inguinal hernia is repositioning the ovary back to pelvis to preserve fertility and repair of inguinal hernia. A multidisciplinary team is required to deal with various abnormalities present in a patient with MURCS syndrome.

## Data Availability

Not available.

## Abbreviations

CXR: Chest X ray
CT: Computed tomography
MRI: Magnetic resonance imaging
MRKH: Mayer-Rokitansky-Küster-Hauser
MURCS: Mullerian agenesis, Renal agenesis, Cervico-thoracic Somite abnormalities
